# Innovations, challenges, and minimal information for standardization of humanized mice

**DOI:** 10.15252/emmm.201708662

**Published:** 2020-06-24

**Authors:** Renata Stripecke, Christian Münz, Jan Jacob Schuringa, Karl‐Dimiter Bissig, Brian Soper, Terrence Meeham, Li‐Chin Yao, James P Di Santo, Michael Brehm, Estefania Rodriguez, Anja Kathrin Wege, Dominique Bonnet, Silvia Guionaud, Kristina E Howard, Scott Kitchen, Florian Klein, Kourosh Saeb‐Parsy, Johannes Sam, Amar Deep Sharma, Andreas Trumpp, Livio Trusolino, Carol Bult, Leonard Shultz

**Affiliations:** ^1^ Regenerative Immune Therapies Applied Hannover Medical School Hannover Germany; ^2^ German Center for Infection Research (DZIF) Hannover Region Germany; ^3^ Viral Immunobiology Institute of Experimental Immunology University of Zurich Zurich Switzerland; ^4^ Department of Hematology University Medical Center Groningen University of Groningen Groningen The Netherlands; ^5^ Department of Pediatrics Duke University Medical Center Durham NC USA; ^6^ The Jackson Laboratory Bar Harbor ME USA; ^7^ Kymab Biotechnology Cambridge UK; ^8^ The Jackson Laboratory Sacramento CA USA; ^9^ Institut Pasteur INSERM U1223 Paris France; ^10^ University of Massachusetts Medical School Worcester MA USA; ^11^ Virology Dept. Bernhard Nocht Institute for Tropical Medicine Hamburg Germany; ^12^ Department of Gynecology and Obstetrics University Cancer Center Regensburg Regensburg Germany; ^13^ The Francis Crick Institute London UK; ^14^ Guionaud Nonclinical Consulting Canterbury UK; ^15^ U.S. Food & Drug Administration Silver Spring MD USA; ^16^ University of California, Los Angeles Los Angeles CA USA; ^17^ University of Cologne Cologne Germany; ^18^ University of Cambridge Cambridge UK; ^19^ Roche Innovation Center Zurich Zurich Switzerland; ^20^ Division of Stem Cells and Cancer German Cancer Research Center (DKFZ) Heidelberg Germany; ^21^ Heidelberg Institute for Stem Cell Technology and Experimental Medicine (HI‐STEM gGmbH) Heidelberg Germany; ^22^ Department of Oncology University of Torino Medical School Turin Italy; ^23^ Candiolo Cancer Institute FPO IRCCS Candiolo Italy

**Keywords:** humanized mice, infections, PDX, immuno‐oncology, regenerative medicine, Chromatin, Epigenetics, Genomics & Functional Genomics, Immunology

## Abstract

Mice xenotransplanted with human cells and/or expressing human gene products (also known as “humanized mice”) recapitulate the human evolutionary specialization and diversity of genotypic and phenotypic traits. These models can provide a relevant *in vivo* context for understanding of human‐specific physiology and pathologies. Humanized mice have advanced toward mainstream preclinical models and are now at the forefront of biomedical research. Here, we considered innovations and challenges regarding the reconstitution of human immunity and human tissues, modeling of human infections and cancer, and the use of humanized mice for testing drugs or regenerative therapy products. As the number of publications exploring different facets of humanized mouse models has steadily increased in past years, it is becoming evident that standardized reporting is needed in the field. Therefore, an international community‐driven resource called “Minimal Information for Standardization of Humanized Mice” (MISHUM) has been created for the purpose of enhancing rigor and reproducibility of studies in the field. Within MISHUM, we propose comprehensive guidelines for reporting critical information generated using humanized mice.

## Graphical abstract

Humanized mice are at the forefront of biomedical research and becoming more mainstream preclinical models. This comprehensive review talks about innovations and challenges regarding the reconstitution of human immunity and introduces “Minimal Information for Standardization of Humanized Mice” (MISHUM).

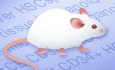

**Glossary**
ADCC: antibody‐dependent cellular cytotoxicity, is an immune defense mechanism whereby effector cells such as NK cells lyses target cells that have been bound by specific antibodies
AML: acute myeloid leukemia
ART: anti‐retroviral therapy
BDBV: Bundibugyo ebolavirus
BiTE: bispecific T‐cell engagers is a registered trademark for a class of recombinant bispecific monoclonal antibodies which bind to the CD3 receptor and to a tumor‐specific antigen
BLT: bone marrow‐liver‐thymus
BM: bone marrow
bNAbs: broadly neutralizing antibodies are antibodies capable of neutralizing different types of viral strains
BRGF: Balb/c *Rag2* ^−/−^ *Il2rg* ^−/−^ *Flt3* ^−/−^
BRGSA2DR2: BRGS mice expressing human HLA‐A2 and DR2 transgenes
CAR: chimeric antigen receptor
CB: cord blood
CCR5: chemokine receptor targeted by R5 tropic HIV strains
CD40L: CD40 ligand
CDX: cell line‐derived xenograft
CRS: cytokine release syndrome is a systemic inflammatory response that can be triggered by infections, drugs, and cell therapies
DCs: dendritic cells
DRAG: mouse strain expressing a human HLA‐DR gene and derived from the NRG strain
EBOV: Zaire ebolavirus
EBV: Epstein–Barr virus
ES cell: embryonic stem cell
*FAH*^−/−^: knock‐out for *the* fumarylacetoacetate hydrolase *gene*
Flt3L: Flt3 ligand
G‐CSF: granulocyte colony‐stimulating factor
GITR: glucocorticoid‐induced TNFR family‐related protein
GM‐CSF: granulocyte–macrophage colony‐stimulating factor
gp: glycoprotein
GVHD: graft‐versus‐host disease
HAdV2: human adenovirus 2
HBV: hepatitis B virus
HCMV: human cytomegalovirus
HCT: HSC transplantation is a routine clinical procedure performed with hematopoietic stem cells from the patient (autologous) or from a donor (allogeneic) with the purpose of combating malignancies or correcting defects of the immune system
HCV: hepatitis C virus
Hematopoiesis: is the differentiation of different blood cell lineages derived from multipotent hematopoietic stem cells (HSCs)
HIS: human immune system
HIV: human immunodeficiency virus
HLAs: human leukocyte antigens
HSCs: human hematopoietic stem cells
HSPCs: hematopoietic/stem/progenitor cells
HSPCs: hematopoietic/stem/progenitor cells
*HSVtk*: herpes simplex virus type 1 thymidine kinase
huPBL: human peripheral blood lymphocyte
IFN: interferon
IgG: immunoglobulin G
IgM: immunoglobulin M
IL‐2: interleukin 2
*Il2rg*: interleukin 2 (IL‐2) receptor common gamma chain
IL‐3: interleukin 3
IO: Immuno‐oncology
iPS cell: induced pluripotent stem cells
LDL: low‐density lypoprotein
Lin^−^: lineage negative
lymphomagenesis: is the development of malignancies derived from lymphocytes such as B and T cells
mAb: monoclonal antibodies
M‐CSF: macrophage colony‐stimulating factors
MDS: myelodysplastic syndrome
MERS: Middle East respiratory syndrome‐related coronavirus
MHC: major histocompatibility complex
MISHUM: minimal information for standardization of humanized mice
MISTRG‐6: mouse strain expressing macrophage colony‐stimulating factors (M‐CSF), IL‐3, IL‐6, GM‐CSF, and thrombopoietin (TPO)
MSCs: mesenchymal stromal cells
Myelo‐ablated mice: are mice treated with irradiation or chemotherapy in order to decrease the bone marrow activity in order to improve the engraftment of transplanted stem cells
Myelodysplasia: is an abnormal accumulation of immature blood cells in the bone marrow
Myelofibrosis: is the replacement of the bone marrow with scar tissue due to proliferation of immature blood cells
NASH: non‐alcoholic steatohepatitis
NIH: National Institutes of Health
NK: natural killer
NOD: Non‐obese diabetic
NOG: NOD.Cg‐*Prkdc* ^*scid*^ *Il2rg* ^*tm1Sug*^/Jic
NRGF: NOD‐*Rag1* ^−/−^ *Il2rg* ^−/−^ *Flk2* ^−/−^
NRG: NOD‐*Rag1* ^*tm1Mom*^ *Il2rg* ^*tm1Wjl*^/SzJ
NSG: NOD.Cg‐*Prkdc* ^*scid*^ *Il2rg* ^*tm1Wjl*^/SzJ
PBMCs: peripheral blood mononuclear cells
PD‐1: programmed death receptor 1
PD‐L1: PD‐1 ligand 1
PDX‐MI: PDX Model Minimal Information standard
PDX: patient‐derived xenograft
PIRF: *POR* ^−/−^/*Il2rg* ^−/−^/*Rag2* ^−/−^/*FAH* ^−/−^
*Rag1*: recombination activating gene 1
*Rag2*: recombination activating gene 2
RESTV: Reston ebolavirus
RSV: Respiratory syncytial virus
SC‐beta: stem cell‐derived human beta cells
SCF: stem cell factor
*scid* mice: CB17‐*Prkdc* ^*scid*^ severely compromised immunodeficient
SCID‐hu: SCID‐humanized
SHIV: simian and human immunodeficiency virus
*Sirpa*: signal regulatory protein alpha
SUDV: Sudan ebolavirus
T1D: type 1 diabetes
TAFV: Tai Forest ebolavirus
TCB: T‐cell bispecific antibodies
TCRs: T‐cell receptors
Tim‐3: T‐cell immunoglobulin and mucin domain‐containing protein 3
TK‐NOG: NOG mice expressing transgenic herpes simplex virus type 1 thymidine kinase (*HSVtk*) under the albumin promoter (NOD.Cg‐*Prkdc* ^*scid*^ *Il2rg* ^*tm1Sug*^Tg(Alb‐Tk)7‐2/ShiJic)
TPO: Thrombopoietin
Tregs: regulatory T cells
TSLP: thymic‐stromal‐cell‐derived lymphopoietin
uPA: urokinase‐type plasminogen activator expressed under the albumin promoter

## Studies of human stem cell engraftment, hematopoiesis, and immunity

Studies using immunocompetent mice have provided critical insights into the development and regulation of hematopoiesis and immunity. However, such studies do not always reflect responses in humans because of multiple species‐specific differences. Therefore, mice developing components of the human immune system (HIS) mice were created. These models have provided tools for the understanding of human hematopoiesis and immunity *in vivo* and to test new therapies or vaccines without incurring risks to patients.

**Figure 1:**
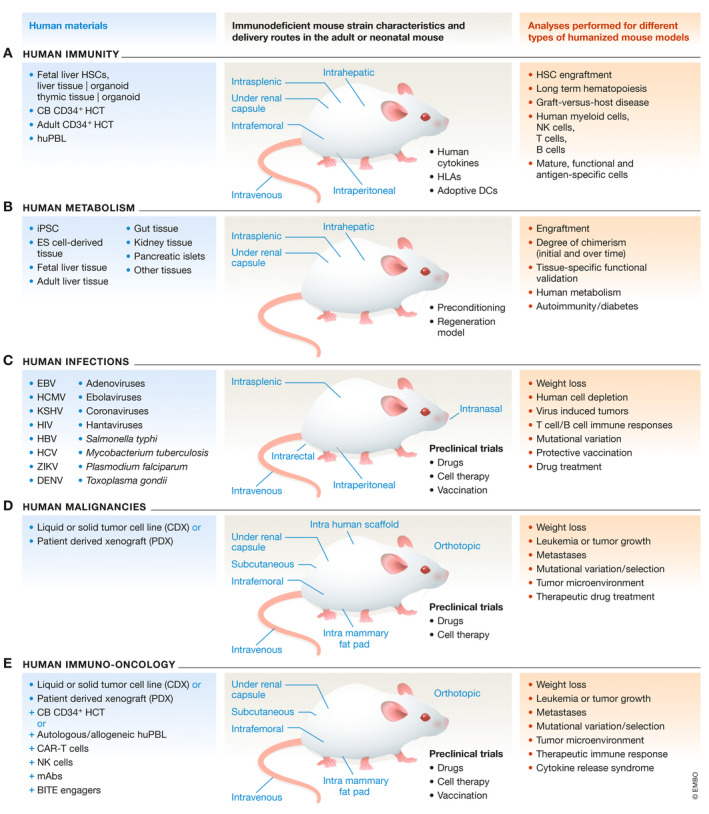
Development and applications of humanized mouse models Schematic representation of the human materials (in blue), immunodeficient mouse strain characteristics and handling (black), and analyses performed (red) for different types of humanized mouse models: (A) human immunity; (B) human metabolism; (C) human infections; (D) human malignancies; (E) human immuno‐oncology. Abbreviated items are spelled out in the glossary.

The simplest engraftment method is the adoptive administration of human peripheral blood mononuclear cells (PBMCs) into severely immunodeficient mice (Fig 1A, Table 1). Since the adoptive human T cells react forcefully against the xenogeneic major histocompatibility complex (MHC) class I and II expressed by mouse tissues, this so‐called “huPBL” model faces the hardship of fulminant xenograft graft‐versus‐host disease (GVHD) occurring 2–4 weeks after PBMC transfer. These models have limited applicability to follow specific antigenic responses, but can be used to test human immunosuppressive agents. Improvement of the huPBL model has been described with novel mouse strains lacking mouse MHC class I and II, resulting in lower occurrences of GVHD (Yaguchi *et al*, 2018; Brehm *et al*, 2019).

**Table 1:** Checklist as a guideline for reporting the “Minimal Information for Standardization of Humanized Mice” (MISHUM)

MISHUM Section 1: human donor
• *Ethical approval and informed consent • *(Gestational) age • Sex • Ethnic origin • Human leukocyte antigens (HLA‐A, B, C, DR) • Known latent viral infections (EBV, HCMV, HIV, HCV, LCMV, HBV) • Exome sequence if available
MISHUM Section 2: human cells or tissues
• *Cell lines (mycoplasma tested or other tests) • *Cell lines or primary cells/tissue available through academic collections and material transfer agreement/publicly available through commercial repositories • *huPBL: Whole blood, PBMC • *HSC: obtained from fetal liver, cord blood, G‐CSF mobilized adult donors, bone marrow • Hepatocytes (±non parenchymal cells) • Primary patient tumors (isolation or collection method) • *Density fractionation (e.g., by Ficoll) • *Surface markers for positive cell isolation (magnetic beads or sorting) • *Surface markers for cell depletions (magnetic beads or sorting) • *Single donor or pooled • *Fresh or cryopreserved • *Dose as viable cell numbers • * Dose of tissue by weight • *Genetic modifications • *Genetic reprogramming (e.g., iPSC) • *Ex vivo expansion • *Ex vivo activation • *Use of scaffolds for 3D culture • *Organoids • Known if latently infected with pathogens
MISHUM Section 3: mouse recipient
• *Institutional approval and approval number • *Strain/source/publicly available or material transfer agreement/stock number • *Human transgenes/knock‐in • *Knock‐out of mouse genes • *Sex • *Age (weeks) • Health reports • Microbiota
MISHUM Section 4: mouse handling
• *Anesthesia (local, general, type and dose) • *Preconditioning (radiation dose/schedule for pharmacologic myeloablation or liver cell death) • *Route of injections (intravenous, intra‐peritoneal, intra‐femoral, intra‐liver, intra‐splenic) • *Surgical implantation (under kidney capsule, intradermal, in mammary fat pad) • *Collection of blood (intravenous, facial vein, cardiac puncture) • *Administration of recombinant cytokines (vendor, units per weight, route) • *Administration of vectors (type, dose, route) • Non‐invasive optical imaging methods (fluorescence, bioluminescence substrate, dose, imaging time, region of interest)
MISHUM Section 5: human hematopoiesis and immunity
• *Relative human HSC engraftment and chimerism (% huCD45^+^ cells in mouse blood at weeks 10, 15, 20 after HCT showing gating strategies) • Absolute human HSC engraftment and chimerism (absolute numbers of huCD45^+^ cells and muCD45^+^ cells in mouse blood at weeks 10, 15, 20 after HCT showing gating and quantification strategies) • *Kinetics of human lymphocyte development (% huCD45^+^, huCD3^+^, huCD4^+^, huCD8^+^ huCD19^+^ cells in mouse blood at weeks 10, 15, 20 after HCT showing gating strategies) • *Human cytokines or chemokines detectable in plasma at terminal analyses (ELISA, bead array methods with appropriate human control samples) • *Human immunoglobulins detectable in plasma at terminal analyses (ELISA, bead array methods with appropriate human control samples) • Kinetics of human myeloid development (% huCD45^+^, huCD33^+^, huCD11c^+^, huCD11b^+^, huCD14^+^ cells in mouse blood at weeks 6, 10, 15, 20 after HCT showing gating strategies) • Kinetics of human NK development (% huCD45^+^, huNKp46^+^, hu56^+^, huCD16^+^ cells in mouse blood at weeks 6, 10, 15, 20 after HCT showing gating strategies) • Kinetics of human B cell development (% huCD45^+^, huCD19^+^, huCD27^+^, huIgM^+^, huIgG^+^, huIgA^+^, cells in mouse blood at weeks 10, 15, 20 after HCT showing gating strategies) • Terminal analyses of human hematopoietic cells in lymphatic tissues (spleen, bone marrow, thymus, peripheral lymph nodes, mesenteric lymph nodes showing total number of cells recovered by tissue). • Terminal analyses of human hematopoietic cells in organs (liver, lungs, brain, etc.…). • Phenotypic characterization of T cells (naïve, central memory, terminal effector, terminal effector memory) • Antigen‐specific characterization of T cells (ELISpot, intracellular staining of IFN‐γ or TNF‐α, tetramer analyses) • Antigen‐specific characterization of antibodies produced by B cells (ELISA, dot‐plot, antigen binding by flow cytometry) • Analyses of antibody functionality against infections (neutralization) • Immune composition by CyTOF • Gene expression analyses (microarrays, RNAseq)
MISHUM Section 6: regeneration of human tissues
• Liver engraftment of hepatoblast, hepatocytes and stem cell‐derived cells (ES or iPSC protocols), lung, gut, endocrine pancreas, kidney or other tissue • Validation of chimerism in the murine blood (ELISA human albumin other secreted proteins) • Functional validation: exogenous test drugs with known and different human metabolism, viral titers or antigens of human hepatotropic viruses (HBV, HCV, etc.) • Validation of chimerism postmortem by immunostaining (human nuclei or other human‐specific antibodies) • Onset of autoimmunity or diabetes.
MISHUM Section 7: human infections
• *Scientific and informal nomenclature for clinical or laboratory pathogen isolates • *Availability through academic collections with material transfer agreement or publicly available through commercial repositories • Biosafety level containment: BSL‐2, BSL‐3, BSL‐3**, BSL‐4 • *Gene modification or reporter gene • *Route of infection: intravenous, intra‐peritoneal, intranasal, intrarectal, intra‐splenic • *Determination of titer and dose of challenge • *Analyses of infection dissemination by PCR (primers, methods) • *Analyses of infection dissemination by histology (antibodies, methods) • Analyses of pathogenesis (load in different tissues, survival, weight loss, liver enzymes, virus‐induced tumor formation) • Analyses of infected cells (FACS, FISH, IF, PrimeFlow, single‐cell sequencing) • Non‐invasive optical imaging methods (fluorescence, bioluminescence substrate, dose, imaging time, region of interest)
MISHUM Section 8: human oncology and immuno‐oncology
• *Donor (age, sex, HLA type) • Primary human tumor or passaged as xenograft • Isolation or selection method of tumor tissue • *Tumor information (HLA expression level, exome sequencing, mutations) • *Cancer identity and metastasis *in vivo* by histopathological analyses • *Autologous or allogeneic to HSCs used in HIS mice • Characteristics after growth (infiltration and activation of human lymphocytes) • Immune modulation of tumor growth
MISHUM Section 9: preclinical testing of human drugs and vaccines
• *Chemical or commercial name • *Vendor or collaboration agreements • *Dose, route, schedule • Pharmacokinetics and pharmacodynamics • Antibody characteristics for passive vaccination • Characteristics of attenuated viruses, of antigen carrying receptor targeting antibodies, of virus‐like particles, and of recombinant viral vaccine candidates • Human drug metabolism in the liver: degree of humanization upon testing, next‐generation strains with human drug metabolism (PIRF or other). • Detection of AST/ALT (liver damage), cytokine release symptom (cytokine storm)
MISHUM Section 10: testing of human cell therapies
• *Production in laboratory scale, GMP‐like or GMP • *Dose of viable cells • *Route, schedule • Pharmacokinetics and pharmacodynamics • Readouts as described above
MISHUM Section 11: statistical and correlative analyses
• Commercially available statistical software (e.g., *t*‐test, ANOVA, etc.…) • Specialized tests used by professional biostatisticians • Heat‐map analyses • Principal component analyses • Neural network analyses • Isogenic control groups or different donors

Asterisks indicate information that should be required in publications.

A more complex approach covered here in detail is the hematopoietic stem cell transplantation (HCT) of preconditioned immunodeficient mice with human hematopoietic stem cells (HSCs). Despite the full mismatch between the human leukocyte antigens (HLA) expressed on the human hematopoietic cells and the mouse MHC expressed on tissues, HCT leads to “fully” humanized HIS models (Fig 1A, Table 1). Human HSCs can differentiate into multiple human hematopoietic lineages, giving rise to mature leukocytes, including several lineages of the human immune system. Robust engraftment with human hematopoietic and lymphoid cells was pioneered back in 1988 with the description of the CB17‐*Prkdc* ^*scid*^ severely compromised immunodeficient (*scid*) strain engrafted with human fetal liver hematopoietic cells and autologous thymic tissues (McCune *et al*, 1988). This SCID‐humanized (SCID‐hu) system showed initially only a transient presence of human T cells and human immunoglobulin G (IgG) in the circulation. The critical relevance of the strain background for engraftment success of human cells was later appreciated when it was observed that non‐obese diabetic (NOD)‐*scid* mice had a much higher capacity to support human HSC engraftment. This was elucidated to be due to the expression of a human‐like signal regulatory protein alpha (*Sirpa*) allele in the NOD strain, popularly known as the “don't eat me signal”, bypassing phagocytosis of human cells by mouse macrophages (Takenaka *et al*, 2007; Shultz *et al*, 2012). Targeting the interleukin 2 (IL‐2) receptor common gamma chain (*Il2rg*) resulted in the absence of mouse natural killer (NK) cell activity as well as ablation of T and B lymphocyte lineages. In addition, the development of mice lacking the expression of recombination activating gene 1 (*Rag1*)^−/−^ and *Rag2* ^−/−^ provided radioresistant mouse models lacking mature host T cells as well as B cells (Shultz *et al*, 2007). Currently, there are approximately 50 diverse humanized mouse models available from biorepositories. Most of these models are homozygous for the *scid*,* Il2rg*,* Rag1*, or *Rag2* mutations and express the NOD or human *Sirpa* allele. The NOD‐*scid IL2rg* ^*(*−/−*)*^ (NSG), the NOD‐*Rag1* ^−/−^ *IL2rg* ^−/−^ (NRG), and the NOD/Shi‐*scid IL2rg* ^*(*−/−*)*^ (NOG) are broadly used strains for xenografting a large variety of human cells, but several other strains are prospering (for recent reviews, see Shultz *et al*, 2019; Allen *et al*, 2019).

It is important to be thoughtful also about the nature of the human HSCs. Although humans and mice differ greatly in their biological characteristics, human HSCs can essentially engraft in myelo‐ablated or irradiated mice and reside in the mouse bone marrow (BM) niche. This HCT approach opened several doors for the understanding of the basic properties for long‐term durable repopulation of human HSCs. As sources of human HSCs, cord blood (CB) or fetal liver are mostly used, as they have high frequencies of HSCs. Generally, a range of 1 × 10^4^–10^5^ isolated HSCs is administered per mouse in order to enable efficient human hematopoietic engraftment and long‐term reconstitution. Several laboratories have opted to use fetal tissues due to the higher abundance in the numbers of HSCs, which can be explored to generate larger cohorts of humanized mice (*n* = 30–40) compared with cord blood (*n* = 10–20). Some groups have tried to overcome this limitation by pooling HSCs from several donors, but upon development of immune systems that are not HLA‐matched, once the T cells develop, allograft reactions among donors can complicate the analyses of the immune responses. Additionally, it is important to take into consideration that HSCs in fetal and neonatal tissues may be intrinsically different regarding the stage of the hematopoietic development. Further, it is important to consider ethical constraints and difficulties in procurement of human fetal tissues. In fact, the US National Institutes of Health (NIH) is currently supporting investigators to seek and develop humanized mouse models that do not rely on human fetal tissues (Allen *et al*, 2019).

Human HSC cell surface markers have been used to allow their identification, purification, and analyses, in order to define the HSC populations with highest engraftment and/or repopulation capacity. Xenotransplantation of human CD34^+^ HSCs into preconditioned immunodeficient mice is the most broadly used procedure to generate HIS mice, and this approach is corroborated by the clinical evidence that transplantation with human‐enriched CD34^+^ hematopoietic/stem/progenitor cells (HSPCs) is a salvage procedure when the HLA is not optimally matched between patients and donors. Remarkably, a defined CD93^hi^ sub‐fraction within the lineage negative (Lin^−^) CD34^−^ CD38^−^ cell present in CB has high repopulating capacity in NOD‐*scid* mice (Danet *et al*, 2002). CD49f is an adhesion molecule serving as a HSC marker and intra‐femoral injection of single CD49f^+^ cells into female NSG mice can generate long‐term (20 weeks) multilineage grafts (Notta *et al*, 2011). Thus, the quest for the archetypical human HSC population and whether other defined CD34^−^ HSCs subpopulations should also be considered for the generation of HIS mice and how to eventually expand these cells *ex vivo* without compromising their self‐renewal potential remains to be clarified. Another aspect to be taken in account is that the ability of human HSPCs to engraft and differentiate into different hematopoietic lineages may largely depend on their interactions with the mouse BM microenvironment constituents. As some human factors may be absent in the mouse BM niche, sponge scaffolds seeded with human BM‐derived mesenchymal stromal cells (MSCs) have been implanted subcutaneously into NSG mice to allow the formation of niches for human HSCs to differentiate (Antonelli *et al*, 2016; Reinisch *et al*, 2016; Abarrategi *et al*, 2017). Using two‐photon microscopy for high‐resolution non‐invasive *in vivo* analyses, these implants are currently enabling the clarification of the human BM microenvironment requirements in regulating human normal and malignant hematopoiesis *in vivo* (Passaro *et al*, 2017).

Another limitation in HIS models is the lack or low levels of human factors and cytokines in mouse tissues or circulating in the plasma and needed for human HSC self‐renewal or differentiation. Transgenic expression of human interleukin 3 (IL‐3)/granulocyte macrophage–colony‐stimulating factors (GM‐CSF)/stem cell factor (SCF) in NSG mice resulted in enhanced levels of human myeloid cells and regulatory T cells (*T*_reg_) (Billerbeck *et al*, 2011). Very promising models are HIS mice generated with a mouse strain expressing several human cytokines such as macrophage colony‐stimulating factors (M‐CSF), IL‐3, IL‐6, GM‐CSF, and thrombopoietin (TPO), the “MISTRG‐6”, and showing improved human T, B, and NK cell development (Das *et al*, 2016; Yu *et al*, 2017). This is a valid approach, and expression of several different human growth factors and cytokines to support differentiation of early or mature lymphoid or myeloid cells has been performed (Rongvaux *et al*, 2014; Bryce *et al*, 2016; Jangalwe *et al*, 2016). Some recent development was also exemplified by transgenic expression of human thymic‐stromal‐cell‐derived lymphopoietin (TSLP) that supported lymph node development in immunodeficient mice (Li *et al*, 2018). Dendritic cells (DCs) are main orchestrators of the adaptive immune system presenting processed peptide antigens to T cells through MHC classes I/II and expressing key costimulatory molecules such as CD40 ligand (CD40L) required for B‐cell activation and class switching (Steinman, 2012). Different types of DCs exist in mice but they are not homologous to human DCs. Further, in HIS mice, the human DC development and maturation are not optimal. Novel HIS models based on the BALB/c *Rag2* ^*(*−/−*)*^ *Il2rg* ^*(*−/−*)*^ *Flt3* ^*(*−/−*)*^ (BRGF) and NOD.Cg‐*Rag1* ^*tm1Mom*^ *Il2rg* ^*tm1Wjl*/*SzJ*^ *Flk2*/*Flt3* ^−/−^ (NRGF) mice with a mutated receptor tyrosine kinase Flk2/Flt3 were created. Human DC development is increased in BRGF and NRGF mice with exogenous administration of human Flt3 ligand (Flt3L) after HCT, leading to a major increase also in the numbers of human NK and T cells (Li *et al*, 2016; Douam *et al*, 2018).

A factor to be taken into account for the generation of HIS is the gender of the mice. Females show better HSC engraftment and faster human T‐cell immune development and maturation (Volk *et al*, 2017). When setting up these models, it is important to keep in mind that the kinetics of human immune reconstitution is not linear and the time of analyses after HCT has to be longitudinally established for different strains and methods. For example, human T cells show maturation, activation, and functionality at 15–20 weeks after CB‐HCT in NRG‐HIS mice (Volk *et al*, 2017; Theobald *et al*, 2018), but this varies considerably for other HIS models. Future improvements are seeking a better development of human T cells in HIS mice so that they will be equipped with functional T‐cell receptors (TCRs) able to interact with the matched HLA complexes on antigen‐presenting cells. This critical advance relies essentially on the substitution of the mouse MHC class I and II by different HLA haplotypes. To solve this mismatch problem, a transgenic NRG mouse strain called “DRAG” was developed that expresses HLA‐DR4 (Kim *et al*, 2017). DRAG mice transplanted with HLA‐DR4^+^ HSCs developed more CD4^+^ T cells and higher levels of human immunoglobulins G and M (IgM and IgG; Kim *et al*, 2017). HSC‐humanized mice expressing class II HLA‐DR4 and class I HLA‐A2 transgenes (“DRAGA” mice) generated CD8^+^‐specific T cells and influenza‐specific antibody responses (Mendoza *et al*, 2018a). Similarly humanized BRGS mice expressing human HLA‐A2 and DR2 transgenes (BRGSA2DR2) showed faster development of CD4^+^ and CD8^+^ T cells and higher concentration of IgGs in plasma (Masse‐Ranson *et al*, 2019). The practical limitation of these HLA‐transgenic strains is that it is difficult to find HSCs that express a particular combination of HLAs.

Another method to improve and accelerate the regeneration of human T and B cells in HIS mice is the adoptive transfer of gene‐engineered human DCs from the HSC donor that are long‐lived *in vivo* (Salguero *et al*, 2014; Daenthanasanmak *et al*, 2015; Volk *et al*, 2017). This approach significantly enhanced the regeneration of lymph nodes in HIS‐NRG mice promoting maturation of functional human T cells, B cell class switching, and development of antigen‐specific IgGs (Salguero *et al*, 2014; Daenthanasanmak *et al*, 2015).

As a take‐home message, development of human immunity in HIS mice depends on several variables (Fig 1A, Table 1). Different approaches are being taken concurrently to accelerate and optimize human immune responses in mice. A structured approach to converge the reporting in scientific publications of the materials and methods (such as specific mouse strains, sex of the mice, methods used for HCT, time‐points of analyses) will facilitate the interactions in the community to boost these promising preclinical models (Table 1).

## Transplantation and regeneration of different tissues to study human metabolism

The liver is a vital organ responsible for key metabolic functions of the body and the site for several human‐specific viral infections. For efficient generation of mice xenografted with human liver tissues, a combination of a growth disadvantage of the murine liver and a regeneration stimulus for the human cells is required. Several approaches resulted in high human liver chimerism in mice (Dandri *et al*, 2001; Mercer *et al*, 2001; Bissig *et al*, 2007; Hasegawa *et al*, 2011) and the resulting models have pros and cons (reviewed in ref. Bissig *et al*, 2018). For example, the transgenic uPA (urokinase‐type plasminogen activator expressed under the albumin promoter) mouse has a profound dysfunction and triggers apoptosis of murine hepatocytes (Heckel *et al*, 1990; Dandri *et al*, 2001; Mercer *et al*, 2001). Therefore, salvage human hepatocyte transplantation is required within 2–4 weeks after birth. Nevertheless, humanized uPA mice maintain considerable health problems. Transgenic uPA mice are difficult to breed, which is also a limitation of another utilized mouse strain based on NOG mice expressing transgenic herpes simplex virus type 1 thymidine kinase (*HSVtk*) under the albumin promoter (TK‐NOG; Hasegawa *et al*, 2011). Conversely, the metabolic dysfunction of the *Il2rg* ^−/−^ *Rag2* ^−/−^ mice with a knock‐out for the fumarylacetoacetate hydrolase *gene* (*FAH* ^−/−^) can be regulated by a small drug and bred efficiently but the mice frequently develop murine hepatocellular cancer (Azuma *et al*, 2007; Bissig *et al*, 2007). All these models show some remaining mouse liver tissue that can blur human‐specific liver metabolism (Fig 1B, Table 1). Therefore, next‐generation models seek to eliminate the interfering mouse metabolism. One such model is the *POR* ^−/−^ *Il2rg* ^−/−^ *Rag2* ^−/−^ *FAH* ^−/−^ (PIRF) mouse (Barzi *et al*, 2017), lacking murine P450 cytochrome function and allowing a human‐only cytochrome metabolism in mice.

Human liver chimeric mice have also been used to model metabolic disorders. The first xenograft model for metabolic liver disease was established using human hepatocytes from a patient with familial hypercholesterolemia with a low‐density lipoprotein (LDL) receptor deficiency; Bissig‐Choisat *et al*, 2015). It would be desirable to extend metabolic disease models also to more prevalent disorders such as non‐alcoholic steatohepatitis (NASH). A NASH model would also require a functional immune system in addition to the liver chimerism. Such dual humanizations have been achieved previously (Gutti *et al*, 2014; Strick‐Marchand *et al*, 2015; Billerbeck *et al*, 2016; Dagur *et al*, 2018). The combined human liver and immune system models can show formation of fibrosis upon hepatitis C virus (HCV) or hepatitis B virus (HBV) infections (Washburn *et al*, 2011; Bility *et al*, 2014).

Another promising approach to study human liver function is the combination of organoid technology with humanized mouse models to examine the immune response to regenerative cellular therapies and cancer. Organoid technology allows the generation of unlimited numbers of non‐malignant (Sampaziotis *et al*, 2017) or cancer cells (Broutier *et al*, 2017; Fig 1B, Table 1). If derived from the same HSC donor used to humanize the mice, this approach can potentially be used to compare the immunogenicity of autologous and allogeneic cellular therapies or investigation of safety and efficacy of autologous cancer immunotherapies.

Studies of type 1 diabetes (T1D) are also prospering with the use of humanized mice. Backcrossing the insulin 2 (Akita) mutation into NRG mice (NRG‐Akita) followed by human HCT into newborn mice resulted in > 50% of the NRG‐Akita mice rejecting human islet allografts (Brehm *et al*, 2010; Fig 1B, Table 1). The Akita model was also used to demonstrate the efficacy of stem cell‐derived human beta cells (SC‐beta) to regulate blood glucose levels *in vivo*. The effect of viral infections was also established for studies of T1D, showing that coxsackievirus B accelerated the destruction of insulin‐producing beta cells of pancreatic islets (Gallagher *et al*, 2015). HIS models are currently being developed to recapitulate the course of disease in human T1D, including the interactions between human immune system and beta cells (Tan *et al*, 2017; Walsh *et al*, 2017).

Recent studies have included engraftment of mice with diverse hematopoietic and non‐hematopoietic human tissues and cell populations, human‐induced pluripotent (iPS) stem cells and embryonic stem (ES) cell‐derived tissues (Shultz *et al*, 2014). Exciting advances on the development and use of emerging humanized mouse models in multiple disciplines are ongoing (Fig 1B, Table 1). Technical and analytical annotation and standardization strategies to harmonize the use of human tissues implanted into humanized mice will be needed (Table 1).

## Infections with human‐specific pathogens

HIS mice offer a unique possibility to study infectious disease agents with a tropism toward human leukocytes, hepatocytes, and lung epithelia, to characterize the induced immune responses and to develop therapeutic interventions against associated pathologies (Fig 1C, Table 1).

One such pathogen is the Epstein–Barr virus (EBV), a common γ‐herpesvirus that persistently infects more than 95% of the human adult population and was the first oncogenic virus identified in man (Münz, 2019). Accordingly, it is associated with around 2% of all malignancies in humans (Cohen *et al*, 2011). Despite the threat of primarily B and epithelial cell transformation in infected individuals, EBV remains asymptomatic in most carriers, presumably due to a near perfect immune control of the virus by cytotoxic lymphocytes (Taylor *et al*, 2015). HIS mice can model this cell‐mediated immune control by primarily CD8^+^ T lymphocytes (McHugh *et al*, 2019). EBV infection resulted in dramatic CD8^+^ T‐cell expansion in humanized mice with a peculiar phenotype (Chatterjee *et al*, 2019; Danisch *et al*, 2019). The expanding CD8^+^ T cells carried the programmed death receptor (PD)‐1 and T‐cell immunoglobulin and mucin domain‐containing protein 3 (Tim‐3) but retained cytokine production and were even superior in cytotoxicity to PD‐1 negative CD8^+^ T‐cell populations. Nonetheless, PD‐1 blockade with antibodies did not improve EBV‐specific immune control in HIS mice (Chatterjee *et al*, 2019). In contrast, PD‐1 inhibition led to elevated EBV titers, increased IL‐10 production, and associated lymphomagenesis. Long‐term infection of HIS mice with another herpes virus, the human cytomegalovirus (HCMV) is also possible as human CD34^+^ cell serves as a latent reservoir, whereas lytic reactivation in monocytes and macrophages can be stimulated with granulocyte‐colony‐stimulating factor (G‐CSF) (for a review, see Koenig *et al*, 2020). HCMV infection and reactivation result in different immunological responses and reactivation is associated with a higher PD‐1 expression on T cells (Theobald *et al*, 2018).

Other pathogens that challenge immune compromised humans, especially pediatric patients after HCT, are human adenoviruses. Human adenovirus 2 (HAdV2) infection of HIS mice resulted in liver pathology in one‐third of mice, while two‐thirds of infected mice remained asymptomatic (Rodriguez *et al*, 2017). HIS mice with asymptomatic HAdV2 infection developed virus‐specific IgM and interferon (IFN)‐γ‐producing T‐cell responses. In blood and BM of mice not showing pathology, only early viral RNA transcripts could be detected, which suggested the establishment of a persistent infection. In contrast, severely affected mice showed both early and late transcripts in many tissues as well as virus production in the liver (Rodriguez *et al*, 2017), all signs of disseminated disease, similar to what is observed in HCT patients that suffer severe HAdV infections (Lion, 2014).

Viruses for which dichotomous outcomes of infection can be modeled in HIS mice are the filoviruses of the genus Ebolavirus (Escudero‐Perez *et al*, 2019). Zaire ebolavirus (EBOV) causes more than 90% mortality after intra‐peritoneal and intranasal infection in HIS mice (Ludtke *et al*, 2015; Escudero‐Perez *et al*, 2019). Sudan ebolavirus (SUDV) causes 70%, Bundibugyo ebolavirus (BDBV) 30%, Tai Forest (TAFV) 20%, and Reston ebolavirus (RESTV) 20% mortality (Escudero‐Perez *et al*, 2019). Immunopathology with pro‐inflammatory cytokines driven by elevated viral loads in the liver was observed in the HIS mice that succumbed to RESTV infection (Escudero‐Perez *et al*, 2019). Thus, HIS mice recapitulate human susceptibility to five of the six known ebolaviruses.

HIS mice also offer platforms to explore new therapeutic avenues. This has primarily been investigated for human immunodeficiency virus (HIV; Marsden & Zack, 2017). For example, treatment of HIV infection with broadly neutralizing antibodies (bNAbs) was established in HIS mice (Klein *et al*, 2012). These studies primarily explored antibodies against four regions of the HIV envelope protein that consists of three heterodimers of glycoprotein (gp)41 and gp120 (Caskey *et al*, 2019). These four regions are the CD4 binding site, the V3 loop, the membrane proximal region, and the V1/V2 region. In these bNAb treatment studies, mutational escape from single bNAb treatment of viremic HIS mice was observed, while mixtures of several bNAbs were able to suppress HIV viral titers for several weeks (Klein *et al*, 2012). Based on these successful treatments in HIS mice and control of hybrid simian and human immunodeficiency virus (SHIV) in macaques (Nishimura *et al*, 2017), HIV‐specific bNAbs were also tested in patients with and without prior anti‐retroviral therapy (ART; Caskey *et al*, 2015, 2017). Similar to HIS mice, individual bNAb treatment suppressed HIV viremia only transiently with escape mutation development. Even transfer of two bNAbs only achieved suppression in HIV‐infected individuals after prior ART treatment (Bar‐On *et al*, 2018; Mendoza *et al*, 2018b). Thus, multiple bNAbs probably need to be maintained at sufficient plasma levels to suppress HIV long term in HIS mice and patients.

Improving T cell‐mediated immune control of HIV through gene therapy is another avenue that is explored in HIS mice. Studies fall into two main categories, either improving T‐cell reactivity by HIV‐specific TCR and chimeric antigen receptor (CAR) expression or rendering T cells resistant to HIV infection (Carrillo *et al*, 2017). For improving T‐cell function, HIV‐specific TCRs were introduced in HSCs and could suppress HIV infection (Kitchen *et al*, 2012). As another strategy, in order to target cells replicating HIV and with gp120 surface expression, an extracellular domain of CD4 fused to the CD3ζ signaling domain was expressed in T cells (Zhen *et al*, 2015, 2017). Downregulation of the HIV co‐receptor, a chemokine receptor targeted by R5 tropic HIV strains (CCR5), has primarily been explored. HIV infection in HIS mice could be significantly compromised by CCR5 deletion or downregulation by zinc finger nuclease‐mediated gene editing or RNA silencing, respectively (Holt *et al*, 2010; Myburgh *et al*, 2015; Shimizu *et al*, 2015). The long‐term survival of such engineered T cells might potentiate a functional cure of HIV, but also raises concerns with respect to toxicities and adverse effects of the respective cellular products, which can be assessed in HIS mice.

Engraftment of additional human tissues, like liver, bone, lung, and thymus, has been reported, but mainly hepatocytes and human lung tissue have been explored for infections with human pathogens. For example, humanized liver mouse models have been used to study infections by different hepatitis viruses (Dandri *et al*, 2001; Mercer *et al*, 2001; Bissig *et al*, 2010; Lutgehetmann *et al*, 2012; Allweiss *et al*, 2016). However, simultaneous reconstitution of human tissues with autologous human immune cells remains a challenge. Nevertheless, HBV and HCV infections have been explored in HIS mice with allogeneic or autologous hepatocyte engraftment (Washburn *et al*, 2011; Billerbeck *et al*, 2016; Dusseaux *et al*, 2017). More recently, bone marrow, liver, and thymus (BLT) engrafted mice have also been combined with ectopic human lung transplants (Wahl *et al*, 2019). Intra‐organ infection of these animals with different human‐specific viruses (Middle East respiratory syndrome‐related coronavirus (MERS), Zika virus, respiratory syncytial virus (RSV), and HCMV) showed virus replication within lung implants, as well as antigen‐specific humoral and T‐cell responses. Further studies in this direction are necessary to widen the application of humanized mice to additional human pathogens and immune responses against them.

These examples of the use of humanized mice to recapitulate human infections and associated immune responses illustrate the vigor and translational value of these models. Standardization of the reporting will improve the interpretation of results for single infections and for cross‐reference among the different pathogens studied (Table 1).

## Cell line‐ and patient‐derived xenografts for human oncology research

Over the past decades, mouse xenograft models have significantly contributed to a better understanding of human malignancies. Cancer‐derived immortalized cell lines can adapt to *in vitro* growth and do not replicate the original malignant physiology seen in patients, potentially leading to artifacts in oncology studies. Thus, patient‐derived xenograft (PDX) models are currently the state‐of‐the‐art approach. Development of liquid and solid PDX models relies on the availability of material obtained from patients with defined types of cancer, which after minimal manipulation is transferred by several routes into immunodeficient mice (Fig 1D, Table 1). Nevertheless, whereas cell line‐based xenografts allow an easier standardization of models, PDX samples are highly variable, can adapt to the murine environment and the human tumor stroma can be eventually replaced by murine cells.

For leukemia research, while the generation of immunodeficient mouse strains like NSG has enabled functional *in vivo* studies on human hematopoiesis, engraftment of (in particular) myeloid malignant cells has remained challenging. The absence of a human bone marrow niche and species‐specific growth factors underlies these challenges, and most notably, it has been difficult to maintain self‐renewal properties of malignant stem cells. Since these populations are thought to be the therapy‐resistant cells that frequently cause relapse of disease, it is of critical importance to use xenograft models in which specifically these cells can be propagated and stemness maintained. In order to further humanize xenograft models, transgenic and knock‐in strains have been generated that (over)express growth factors like IL‐3, GM‐CSF, SCF, TPO, and/or M‐CSF, as well as others (Wunderlich *et al*, 2010; Rongvaux *et al*, 2014). An alternative approach has been to develop a human microenvironment in the mouse initiated by mesenchymal stem cells coated on 3D scaffolds (Antonelli *et al*, 2016; Abarrategi *et al*, 2017; Carretta *et al*, 2017) or embedded in matrigel (Reinisch *et al*, 2016). These models have allowed the engraftment of various hematological malignancies, including those that are notoriously difficult to engraft in regular NSG models such as low‐risk acute myeloid leukemia (AML), myelodysplastic syndrome (MDS), and myelofibrosis. Importantly, self‐renewal was better maintained in these models as shown by serial transplantation experiments and transcriptome studies. As such, these models more faithfully capture the disease phenotypes as seen in human patients and therefore are likely to produce more clinically relevant and translatable results when used in drug screens. A challenge that remains is that myeloid malignancies, in particular AML, display a complex clonal heterogeneity. Multiple genetically distinct subclones can co‐exist within an individual patient, each driven by a similar founder mutation but with different secondary driver mutations. These clones are not only genetically distinct; they also differ remarkably at the transcriptome, epigenome, and cell biological level (de Boer *et al*, 2018). To develop curative therapies, this clonal heterogeneity needs to be taken into account. It has become clear that not all clones of an individual patient might engraft equally efficiently in mice, and also the level of humanization of the model used might impact on whether the true clonal heterogeneity is preserved *in vivo* (Klco *et al*, 2014; Antonelli *et al*, 2016; Carretta *et al*, 2017; Wang *et al*, 2017; de Boer *et al*, 2018). Both (deep) sequencing technologies and flow cytometry‐based approaches are useful tools to dissect clonal heterogeneity, *in vitro* as well as in mouse models. For instance, an “infinicyt”‐based approach, which combines expression profiles of multiple aberrant AML‐specific plasma membrane proteins, can provide subclone‐specific insights into the clonal complexity of the malignancy under study (de Boer *et al*, 2018). Implementation of such technologies is warranted in any conducted *in vivo* xenograft experiment to link drug responses to specific genetic features of malignant clones.

In the case of solid tumors, the use of PDXs for preclinical drug development holds potential to improve our knowledge of the principles underlying responsiveness to individualized treatment regimens (Hidalgo *et al*, 2014; Byrne *et al*, 2017). Yet, many questions are still open, in particular concerning the ability of the PDX approach to directly influence clinical decision making (Aparicio *et al*, 2015). Not all cells that compose the parental tumor successfully engraft in the mouse, which introduces a selective pressure for genetic variants conferring better survival fitness (Ben‐David *et al*, 2017). The subsequent propagation steps may also affect clonal dynamics, with further deviation of serially passaged samples from the primary tumor from which they were derived (Eirew *et al*, 2015). The lack of a fully functional immune system in the host and the fact that human stromal components—such as cancer‐associated fibroblasts, endothelial cells, and inflammatory cells—are replaced by murine counterparts add extra layers of divergence over native tumors (Hidalgo *et al*, 2014; Aparicio *et al*, 2015; Byrne *et al*, 2017). These limitations notwithstanding PDX models of solid tumors offer considerable opportunities for biomarker and target nomination. First, although serially passaged PDXs are likely to be genetically different from the matched tumor of their donor patients, they are expected to display genomic makeups and polyclonality patterns that, on a probabilistic basis, may be similar to those of tumors that spontaneously develop in unrelated individuals (Eirew *et al*, 2015; Byrne *et al*, 2017). These factors make PDXs critical tools in the translational oncology domain, whereby predictive biomarkers discovered in PDXs may be leveraged for the prospective identification of patients with tumors exhibiting the same biomarker repertoire. Second, responses to therapies that target driver oncoproteins are thought to be only partly influenced by microenvironmental parameters and more directly dependent on cancer cell‐intrinsic features, which affords results in PDXs with adequate predictive power for cancer cell‐directed treatments (Byrne *et al*, 2017). Finally, vast PDX collections are poised to capture inter‐patient tumor diversity on a population scale, thus representing powerful platforms for large‐scale genotype–response associations (Gao *et al*, 2015).

The above examples illustrated the importance of PDX models in understanding the evolution of tumor growth, investigating the mechanisms of drug resistance, and developing personalized treatments. Critical to these studies is ensuring researchers have access to high‐quality PDX models and molecular datasets that give sufficient power to perform informative analyses. However, the complex nature of PDX models and the heterogeneous resources that generate them often lead to crucial information about tumors, host strains, transplant, and quality assurance processes being inconsistently presented. To address this challenge, the PDX research community developed the PDX Model Minimal Information standard (PDX‐MI) that defines the critical metadata needed to exchange knowledge about PDX models (Meehan *et al*, 2017). PDX‐MI describes the clinical attributes of a patient's tumor, the processes of implantation and passaging of tumors in a host mouse strain, quality assurance methods, and the use of PDX models in cancer research. Since its inception, PDX‐MI has been adopted by producers of PDX models including the international PDXNet and EurOPDX consortia as well as the PDX Finder Catalog that captures, harmonizes, and disseminates data about PDX models and associated Omic datasets (Conte *et al*, 2019). PDX‐MI promotes reuse of models and data, maximizing the impact of these models on oncology research and facilitating the development of new treatments.

## Human immuno‐oncology research

Therapeutic modulation of the human immune system to improve recognition and response to tumors is a clinically accepted revolution in oncology treatment for multiple tumor types (Pardoll, 2012). Immuno‐oncology (IO) is considered a breakthrough due to significant and durable tumor regression coupled with increased long‐term survival. However, these clinical responses only occur in a subset of patients. Therefore, considerable investment in preclinical research is still necessary to identify new and improved approaches to cancer cell‐specific immune response as well as testing of combinatorial strategies. These and other approaches are typically developed in syngeneic mouse models of oncology to work out mechanisms of action. Nonetheless, human‐specific immune modulators require *in vivo* models with human‐specific targets on both human immune cells and human tumors to validate preclinical responses, accelerate development, and improve translation to the clinic. Ideally, IO *in vivo* studies will rely on the combination of HIS and PDX models. As described in the following sections, HIS mice co‐engrafted with human tumors are proving to be a valuable tool in the development of new strategies for human‐specific immuno‐oncology therapies. Nonetheless, as these models are *per se* complex and the matching of the tumor and immune system from the same patient is currently a difficult task, several studies explore cell line derived xenograft (CDX) implanted after humanization in HIS mice or administration of human peripheral blood lymphocytes (PBL; Fig 1E, Table 1). These IO studies have been used, for example, to test immune modulation caused by engineered agonistic or antagonistic monoclonal antibodies (mAb; Scott *et al*, 2012), bispecific T‐cell engagers (BiTE; Baeuerle & Reinhardt, 2009), or T‐cell bispecific antibodies (TCB; Bacac *et al*, 2018).

A very important and commonly asked question regarding human tumor cell engraftment in HIS mice is whether the CDX or PDX tumor and hematopoietic donor must be 100% HLA‐matched to allow co‐engraftment. Neonatal irradiated NSG mice co‐injected into the liver with CD34^+^ cord blood‐derived HSC and HLA‐mismatched human breast CDX, resulted in the development of a human immune system together with human tumor growth, including metastases in the lung and brain (Wege *et al*, 2011, 2014). Tumors were partially infiltrated with T cells, B cells, and myeloid cells. More detailed analyses of the spleen revealed not only a T‐cell specific activation pattern but also B‐cell maturation and the production of tumor‐specific antibodies (Wege *et al*, 2014). Moreover, the tumor engrafted HIS mice were used for a preclinical trial to test the potential of IL‐15 in combination with trastuzumab (anti‐HER2 mAb) therapy with the intention to enhance antibody‐dependent cellular cytotoxicity (ADCC; Wege *et al*, 2017). IL‐15 treatment triggered immune activation and promoted tumor depletion but also induced systemic inflammation, resulting in death of the treated mice (Wege *et al*, 2017).

In another study, 3‐week‐old NSG mice were engrafted with cord blood‐derived CD34^+^ HSC first to establish mature multilineage immune engraftment and then injected subcutaneously 12–16 weeks later with partially HLA‐matched PDX tumors (Wang *et al*, 2017). Despite the presence of a wide range of functional immune cells, the HIS mice were capable of engrafting the tumors and in many cases the growth kinetics of these tumors did not vary significantly from immunodeficient controls not engrafted with HSC. However, not all partially HLA‐matched tumor/HSC donor combinations escape immune‐mediated changes in growth kinetics and some tumors are rejected, highlighting the importance of empirically testing tumor growth against multiple HSC donors. Subcutaneously engrafted tumors were infiltrated with a wide range of human innate and adaptive immune cells and both the frequency and distribution of immune cell types varied across different tumor types (Wang *et al*, 2017). One mechanism known to prevent T cells from responding to tumors is the PD‐1 and its ligand (PD‐L1) checkpoint pathway. The clinically approved checkpoint inhibitor pembrolizumab (anti‐PD‐1 mAb) has been tested in tumor‐bearing HIS mice and suppression of tumor growth was observed using both CDX and PDX tumors (Wang *et al*, 2017). Suppression of tumor growth with pembrolizumab only occurred in mice co‐engrafted with human immune cells and the response was abrogated when mice were pretreated with anti‐human CD8 mAb to deplete human CD8^+^ T cells, demonstrating human CD8^+^ T cells mediated the effector response following release from checkpoint inhibition. Efficacy studies with pembrolizumab were run with multiple HSC donors distributed among both control and treatment arms of each tumor tested for response. Multiple HSC donors allowed the observation that not all tumor/HSC combinations show a response to pembrolizumab, and the frequency of donor‐related response (~ 25–30%) is similar to what is observed in the clinic (Topalian *et al*, 2012).

Regulatory T cells (Tregs) infiltrate a wide range of tumor types and mechanism of action studies performed in syngeneic mouse tumor models revealed that depletion of these cells from the tumor could release T effector cells from Treg suppression (Smyth *et al*, 2014). Preclinical efficacy for this approach was demonstrated when HIS mice were engrafted with SK‐MEL‐5 human melanoma CDX and treated with an anti‐human mAb targeting the glucocorticoid‐induced TNFR family‐related (GITR) protein, highly expressed on Tregs. Tumor growth was significantly suppressed, the percentage of Tregs was reduced in tumor and spleen, and tumor‐infiltrating lymphocytes showed increased secretion of the effector cytokines IL‐2 and IFN‐γ (Mahne *et al*, 2017).

As more IO treatments move through clinical trials, clinicians are seeing an association between strong immune‐mediated tumor killing responses and cytokine release syndrome (CRS). Given these observations, preclinical studies able to recapitulate CRS in HIS mice are becoming highly relevant. In a recent report, HIS mice were co‐engrafted with a diffuse large B‐cell lymphoma (WSU‐DLCL2) and treated with either obinutuzumab (anti‐CD20 mAb) or a novel CD20‐T‐cell bispecific antibody (TCB) containing two CD20 binding domains and one CD3ε domain in a head‐to‐tail orientation to one of the CD20 regions (Bacac *et al*, 2018). CD20‐TCB promoted a more extensive killing response than obinutuzumab. Further, CD20‐TCB administration was associated with increased expression of multiple human inflammatory cytokines indicating a CRS response that was not observed with obinutuzumab treatment. An alternate strategy was tested where treatment was initiated with a single dose of obinutuzumab followed by multiple high doses of CD20‐TCB. The pretreatment with obinutuzumab strategy enabled rapid and extensive tumor killing with minimal CRS response. These types of preclinical experiments with HIS mice demonstrate their value in working out protocols designed to maximize both efficacy and safety.

The question of immuno‐therapy‐mediated toxicity, particularly in the context of CRS, is a key component of preclinical evaluation and a reliable assay is needed. Neither *in vitro* assays nor non‐human primates have proven reliable for assessment of CRS (Stebbings *et al*, 2007). A team at the US Food and Drug Administration recently published two reports testing CRS using mAb therapies known to have a strong cytotoxic response in the BLT‐HIS mice (Yan *et al*, 2019a,b). BLT mice were injected with adalimumab (anti‐TNF‐α mAb) as a negative control because it is used clinically without evidence of CRS. The test article was TGN1412 (anti‐CD28 mAb), a reagent known to be associated with clinical CRS. TGN1412‐treated BLT mice released multiple cytokines into peripheral blood within 2‐4 hours of treatment, indicating a strong CRS response that was not observed in the adalimumab‐treated group (Yan *et al*, 2019a). The mice also showed a decrease in human CD45^+^, CD3^+^, CD4^+^, CD8^+^, and CD19^+^ cells in peripheral blood similar to human patients and showed an increase in murine serum amyloid A, indicating severe liver inflammation. A second study compared muromonab (anti‐CD3 mAb, OKT3) to adalimumab in BLT mice. The muromonab‐treated mice released multiple proinflammatory cytokines associated with CRS into peripheral blood within 2–4 h, and pretreatment of mice with methylprednisolone prior to muromonab blunted or delayed development of CRS (Yan *et al*, 2019b). Together, these studies show that BLT‐HIS mice are capable of recapitulating multiple aspects of a strong CRS response when dosed with mAb therapies designed to stimulate strong T‐cell activation.

To our knowledge, no direct comparisons between PBMC‐HIS and BLT‐HIS model mice have been made to date. There are several published studies using PBMC‐HIS mice demonstrating CRS (Brady *et al*, 2014; Weissmuller *et al*, 2016) with the mice being used between 6 and 16 days of PBMC injection. In both reports, the authors state there were no signs of GVHD present when used. In Yan *et al* (2019a), a limited comparison of CD34‐HIS mice and BLT‐HIS mice was undertaken and BLT‐HIS mice showed clear evidence of CRS and no GVHD, whereas CD34‐HIS mice did not show any difference as compared to control treatment, suggesting that the CD34‐HIS mouse does not show a clear signal for CRS and may not be an appropriate model. As previously published for the BLT‐HIS mouse (Weaver *et al*, 2019), when present, GVHD is clearly evident and can be differentiated from other processes, such as CRS. GVHD occurs rapidly and for all PBMC‐HIS mice, but does not occur with high frequency in BLT‐HIS mice. With respect to CRS, PBMC‐HIS mice would potentially demonstrate CRS for drugs specifically impacting T cells, but not other tissues or cell types. BLT‐HIS mice have much broader engraftment in terms of cell types and presence in non‐lymphoid organs, suggesting that a wider range of targets could demonstrate CRS if present. For preclinical use, the Stebbings *in vitro* assay (Stebbings *et al*, 2007) should be initially undertaken. Studies with HIS mice would be adjunctive and informed by both the target of the therapeutic and what organ system(s) were targeted. Additional circumstances in which *in vivo* testing could be helpful include higher risk drug targets and non‐T‐cell targets.

In conclusion, HIS mice are a powerful tool for IO research. HIS mice do not recapitulate every aspect of human immunity, but they are capable of answering a wide range of important scientific questions that form a critical guide for preclinical IO discovery. Future challenges will include the understanding of the donor‐to‐donor immune variability observed in some of these treatment strategies. This will provide opportunities to identify predictive markers and clinical diagnostics assays helpful in assisting patient enrollment for improved treatment outcomes. Tumor populations that escape response in subsets of mice can be further analyzed for understanding mechanism of resistance.

## Minimal information for standardization of humanized mice

Humanized mouse models result from the sum of several components: choice and availability of human donors, human cells or tissues, mouse recipient, types of manipulations, human infections, and human tumor types. Furthermore, the materials available for analyses and methods of analyses provide another level of complexity. It is quite clear that humanized mouse models are customized by the different laboratories around the globe and it would be unrealistic to standardize how the models should be built. Nevertheless, reporting of minimal information provided by specific guidelines can facilitate independent validation of published data, which is a fundamental cornerstone for scientific advancements. The list of variables provided in Table 1 is an initial attempt brought up by the faculty participating in the 2017 and 2019 EMBO Practical Courses created for training young investigators on the development of humanized mouse models.

This initiative called “Minimal information for standardization of humanized mouse models” (MISHUM) is built on the experience of the authors of this manuscript in developing similar reporting standards for non‐humanized PDX models (Meehan *et al*, 2017). The main aim of this workgroup is, similar to PDX‐MI, to promote material and protocol exchange, transparency in reporting of assays and analyses performed. The standards described here represent a starting point. The longer term intention is to extend this initiative beyond the EMBO courses to include input from multiple stakeholders from both academia and industry. Further, in order to enable a standardized interpretation of already published results, the collection of technical information and quantifiable data can in the future be procured within MISHUM for the creation of a digitalized database. Once these standards and database are evolving, a parallel data mining activity will provide the opportunity to explore and discover convergent signatures and patterns of human immunology, infections, and oncology *in vivo* in humanized mice. Our long‐term goal is that, in the future, these models will become largely reproducible and predictive models for the understanding of human physiology, immunology, and oncology, which require a living experimental system. Beyond the gain of scientific information, we hope to make humanized mouse models more environmentally sustainable by optimizing the methods and reducing the number of humanized mice used for experimentation. Ultimately, we seek to support the “3R” principles: (i) Replace the use of humanized mice if alternative *in vitro* techniques (such by the use of organoids or chips), metadata or data mining eventually prove to be as solid as the *in vivo* results; (ii) reduce the number of humanized mice to a minimum within each experimental cohort; and (iii) refine the experimental setup using the best and ethically available human material and analyses, also making sure that invasive approaches can be minimized to mitigate the suffering of the animals. Only with a consensus checklist and with a coherent reporting policy, we will be able to identify the best material, methods, and analyses that will ultimately lead to optimized humanized mouse models.

## Pending issues

Below is a summary of main topics identified by our community that remain to be solved on a case‐by‐case basis depending on the use of the humanized mouse model:

☐ *Human hematopoiesis and immunity:* Methods to expand human HSCs will enable larger experimental cohorts. Novel methods for matching the HLA between human hematopoietic cells and mouse epithelial cells will improve human T‐cell development in a HLA‐restricted manner. Better development of lymph nodes and germinal centers within the spleen that would improve innate and adaptive immune responses. Novel mouse strains or methods allowing regeneration of lymph nodes will allow B cell class switch and production of human high‐affinity IgG and IgA antibodies.
☐ *Human metabolism*: Mice and human display different rates and pathways of metabolism, and particularly for liver metabolism, it is essential to address this limitation of humanized mice. Since many metabolites are diffusible, new models are needed that can eliminate or temporarily block murine metabolism while using chimeric mice.
☐ *Human infections*: Engraftment of human peripheral tissues (i.e., liver, lung, skin, and brain) will allow infections with human pathogens targeting other tissues than immune cells, and the possibility of combining them with HLAs matched human hematopoietic stem cells will create a more complete model of human infection. Better engraftment of the human erythrocyte lineage will allow further studies of erythrocyte‐infecting pathogens (i.e., plasmodium).
☐ *Human oncology*: Further humanization of xenograft mouse models such as implantation of human MSC‐coated 3D scaffolds or NSG mice (over) expressing human cytokines has improved engraftment rates of primary tumor cells. However, for each individual cancer patient case, it will have to be established which (sub)clones preferably grow out. Also, it will need to be carefully evaluated how the transcriptome and epigenome of the original patient samples are preserved in the PDX models.
☐ *Human immuno‐oncology*: The HLA matching of the human immune system and tumor will be required. A functional enhancement of the CD4^+^ and CD8^+^ T cells will be needed to allow higher tumor infiltration and anti‐tumor responses. An improved and faster development of “avatar” PDX models will allow decisions about personalized therapeutic options.

## Conflict of interest

JAX is a not‐for‐profit organization that commercially distributes several of the mentioned immune‐deficient mouse strains and PDX models, deposited in the repository. BS, LCY, CB, and LS are JAX employees. RS and MB receive research funding from JAX. The other authors declare no competing interests.

## For more information

(i) https://www.embl.de/training/events/2019/HUM19-01/ EMBO Practical course “Humanized mice in Biomedicine: Innovations and Challenges” held in 2019 at the European Molecular Biology Laboratory (EMBL) in Heidelberg, Germany.
(ii) https://www.embl.de/training/events/2017/HUM17-01/index.html EMBO Practical course “Humanized mice in Biomedical Research” held in 2017 at the European Molecular Biology Laboratory (EMBL) in Heidelberg, Germany.
(iii) https://www.mdpi.com/journal/vaccines/special_issues/Humanized_Mice In this Special Issue “Humanized Mice in Vaccinology: Opportunities and Challenges”, aspects related to the use of humanized mice in vaccinology, opportunities, and the challenges ahead are discussed.
(iv) http://www.pdxfinder.org/pdx-standard/ The PDX Minimal Information document represents the results of a broad community effort to develop a standard regarding the essential information needed to describe a PDX model.
